# Three‐dimensional Cherenkov emission surface mapping into the patient coordinate system for spatial delivery verification in EBRT

**DOI:** 10.1002/acm2.70566

**Published:** 2026-05-31

**Authors:** Alexander Geiersbach, Michael Jermyn, David Gladstone, Lesley Jarvis, Brian Pogue, Petr Bruza

**Affiliations:** ^1^ Thayer School of Engineering Dartmouth College Hanover New Hampshire USA; ^2^ Department of Radiation Oncology and Applied Sciences Geisel School of Medicine Dartmouth College Hanover New Hampshire USA

**Keywords:** 3D surface mapping, optical quantitative imaging, photon EBRT

## Abstract

**Background:**

Cherenkov imaging provides the ability to visualize beam delivery during radiation therapy treatments in real‐time informing the therapy team about possible deviations from the treatment plan. Current clinical practice with Cherenkov imaging relies on the real‐time view and post‐treatment qualitative assessment of Cherenkov images by therapists, physicists and clinicians who may then perform secondary treatment checks.

**Purpose:**

While Cherenkov images provide new insights into beam location, the limitation is that quantifying the observed deviation is difficult due to the lack of spatial registration of the Cherenkov emission in patient's coordinate space. This work addresses this problem with the first fusion of Cherenkov images with the three‐dimensional patient surface to enable quantitative spatial beam verification in patient coordinate space. By combining multiple camera viewpoints with CT‐sim derived surface coordinates, this system is capable of generating virtual beam's eye view images for objective measurement of beam delivery deviations on patient surfaces. This approach enables automated error detection thresholds and represents the first step toward real‐time, quantitative optical treatment verification and dosimetry.

**Methods:**

Cherenkov images from multiple geometrically calibrated cameras were projected onto Computed Tomography (CT) surface datasets to provide a Cherenkov surface image map on the patient. A raycasting algorithm was utilized to virtually map the Cherenkov surface image into the beam's eye view. To test the ability to quantitatively measure beam versus patient surface deviations from the virtual beam's eye view, a series of 10 × 10 cm^2^ 6X fields with simulated spatial shifts were delivered to both flat and anthropomorphic phantoms. Virtual beam's eye view images were generated for each shift, and the magnitudes of Cherenkov map shifts were measured with Iterative Closest Point (ICP) and Hausdorff distance metrics with respect to a reference field.

**Results:**

Iterative Closest Point algorithms achieved an average x and y accuracy of 0.5 ± 0.2 mm and 0.3 ± 0.2 mm for the flat phantom and 0.5 mm ± 0.7 mm and 0.3 mm ± 0.2 mm for the anthropomorphic torso phantom. Camera projection error adds up to an additional 0.9 mm of error.

**Conclusion:**

Multi‐view surface mapping of Cherenkov images onto the patient surface mesh enabled quantitative assessment of beam versus topology shifts between fractions, using the Cherenkov emission outline as a beam extent surrogate. We demonstrated the utility and metrics of Cherenkov 3D mapping towards future applications utilizing daily or real‐time surfaces obtained from, for example, cone‐beam CT or surface‐guided radiotherapy (SGRT) systems.

## 1 INTRODUCTION

Cherenkov imaging enables real‐time visualization of beam delivery during external beam radiation therapy (EBRT).^1^ When megavoltage (MV) radiation passes through tissue, secondary electrons exceeding the phase velocity of light generate broadband optical Cherenkov emission, which can be detected by time‐gated intensified cameras. Imaging this emission during treatment reveals the spatial and temporal pattern of each beam as it is delivered. Cumulative images from each fraction can be compared with the planned fluence to detect delivery deviations, enabling correction in subsequent fractions.

Clinical studies in a retrospective cohort of 622 patients^2^ demonstrated that Cherenkov imaging detected otherwise unrecognized, clinically significant incidents in approximately 1.4% of fractions. The technique complements surface guided radiotherapy (SGRT) by adding direct visualization of the radiation field, whereas SGRT alone provides only positional data in patient coordinates.^3^ However, current Cherenkov imaging produces a fixed camera‐space view, limiting geometric interpretation.^4^

Most Cherenkov imaging systems use two oblique camera views, with an optional couch‐foot view, displayed as pseudo‐color Cherenkov emission overlays on grayscale anatomy. These allow visual detection of deviations such as unplanned anatomy in the beam^2^ or bolus misplacement,^5^ but remain spatially qualitative due to the oblique acquisition geometry and the lack of surface geometry reference. Quantitative spatial analysis requires mapping Cherenkov images into patient coordinates, which can be achieved by projecting them onto 3D patient surface meshes. Here, we combine multi‐view Cherenkov imaging data with surface meshes to create the first 3D Cherenkov emission maps, enabling visualization from arbitrary perspectives, including a virtual beam's‐eye view (BEV) showing the delivered field shape on the patient.

This work develops and tests the utility of 3D Cherenkov surface mapping to:

1. Merge multiple Cherenkov imaging viewpoints to avoid incomplete Cherenkov visualization due to gantry and tissue obstructions;
2. Evaluate displacement metrics to measure the Cherenkov surface map deviations compared to Day 1 treatments;
3. Determine the limits of spatial accuracy of combined spatial Cherenkov image mapping.

Here static CT surfaces were used as the source of patient surface meshes and as the first step towards mapping that utilizes real‐time SGRT maps. A raycasting algorithm^6^ was developed to project Cherenkov images from known camera positions onto static CT surface meshes. Textured patient surface meshes were then used to generate parallel and point projections of the Cherenkov emission from beam's eye views for quantitative spatial analysis. Point projection images were used to show users the beam shape on the patient surface, while parallel projection images were used to measure beam deviations.

In this work, Cherenkov camera calibration accuracy was first assessed, then the raycasting accuracy was evaluated on a flat phantom. Next, the surface map coverage was improved by combining two Cherenkov images on an anthropomorphic phantom. Finally, the Iterative Closest Point^7^, ^8^ (ICP) and Hausdorff^7^, ^9^ metrics were quantified on virtual beam's eye view parallel projection images to quantitatively detect and measure beam shifts on the patient surface.

## 2 MATERIALS AND METHODS

### 2.1 Experimental setup

**FIGURE 1:**
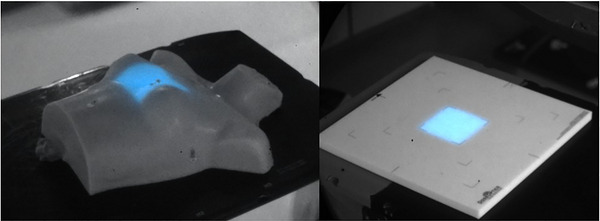
[Phantoms used for Cherenkov surface imaging] Anatomical Annie Phantom (Left) and ABS Phantom (Right) with 10 × 10 cm^2^ fields delivered.

Measurements were taken on two different linear accelerators (Varian Trilogy and TrueBeam, Varian Medical Systems, Palo Alto, CA) in two different therapy vaults at the Dartmouth Cancer Center. 6 MV beams were delivered at a dose rate of 600 monitor units per minute and a source to surface distance (SSD) of 100 cm to simulate clinical whole breast radiotherapy conditions. Each measurement used between 100 and 300 MU to maximize signal in each image set. Standard field sizes of 10 × 10 cm^2^ were delivered to each phantom (see Figure 1).

Images were taken with two sets of clinical Cherenkov cameras (BeamSite, DoseOptics, Lebanon, NH) mounted inside each of the two treatment vaults. Both sets of Cherenkov cameras use fixed focal length lenses (50 mm f/1.2). Imaging systems were calibrated using factory isocenter calibration and positional calibration, providing camera position coordinates, focal direction vectors, view up vectors, and view angles.

A flat ABS plastic slab (40 cm × 40 cm × 1 cm, DoseOptics) phantom and a tissue‐mimicking anthropomorphic silicone phantom^10^ were used as delivery targets. Both phantoms were set up at the isocenter in the same position as their respective CT scans.

The flat phantom was set up with its center at the isocenter (100 cm SSD) and used for virtual beam's eye view imaging, as its planar shape allowed for easy validation of projection models. The anthropomorphic phantom served to simulate a typical clinical case with more complex geometry than the flat phantom. This phantom was set up at isocenter with respect to positional radio‐opaque markers with an isocenter inside the phantom (97 cm SSD). A computer tomography (CT) scan was taken with 0.6 mm/slice resolution, and a stereolithographic STL surface mesh file was extracted from DICOM datasets with simple thresholding.

### 2.2 Algorithm for surface mapping

**FIGURE 2:**
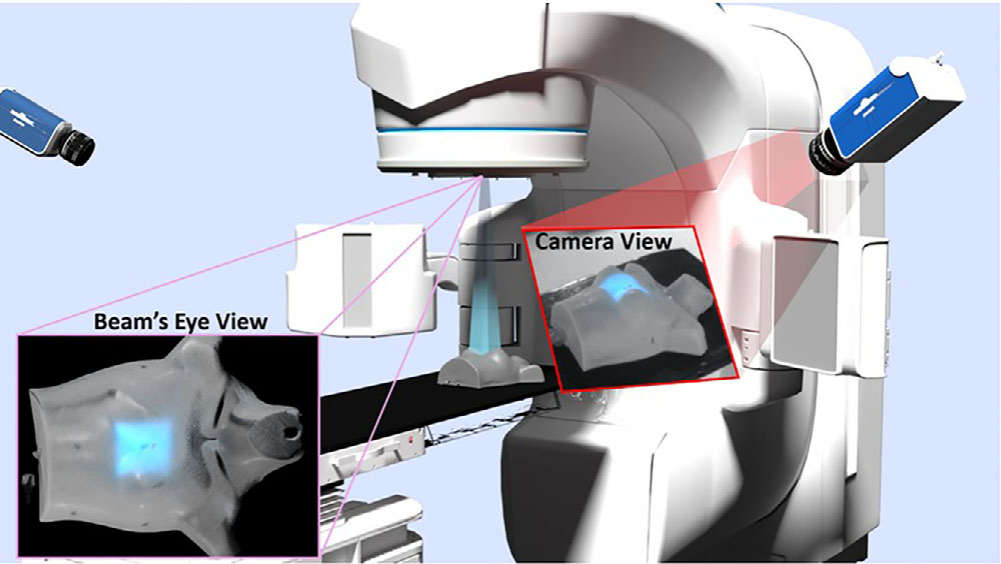
[Raycasting overview] Transformation of multiple side view Cherenkov images fused to create one virtual beam's eye view image.

To integrate Cherenkov imaging with the patient's 3D geometry, the recorded camera images must be accurately mapped onto the patient surface mesh. This was accomplished using a raycasting approach, in which each pixel in the Cherenkov image is traced along a ray from the camera toward the 3D model. When a ray intersects the patient surface, the corresponding Cherenkov intensity value is assigned to that point on the mesh. Applying this process to all pixels generates a surface texture that represents the spatial distribution of Cherenkov emission on the patient. Once textured, the surface mesh can be virtually re‐rendered from any viewpoint, including a beam's‐eye view from the treatment source position (See Figure 2).

The raycasting process required three essential data structures: (1) Cherenkov images, (2) Cherenkov camera spatial calibration data, and (3) patient surface data.

1. Patient (phantom) surface data were acquired using a Siemens SOMATOM CT scanner. This data were exported from the Eclipse treatment planning software in Aria using the DICOM export media filter. Body contours were converted to STL format using 3D Slicer.
2. Cherenkov camera positioning data were acquired using BeamSite calibration protocols and is stored as a text file alongside Cherenkov images after each image acquisition. The following essential data about the camera position is included in the extrinsic calibration file: Camera position in mm (x_1_, y_1_, z_1_), focal position in arbitrary units (x_2_, y_2_, z_2_), view up vector in arbitrary units (x_3_, y_3_, z_3_), and the view angle in degrees.
3. Cherenkov images were acquired using BeamSite software. Images were captured at a framerate of 17 fps, internally darkfield‐corrected and background‐subtracted, and cumulative images were produced at a resolution of 1920 × 1200 pixels with 32‐bit depth. These images were captured as raw 32‐bit frames and converted to png format using ImageJ.

**FIGURE 3:**
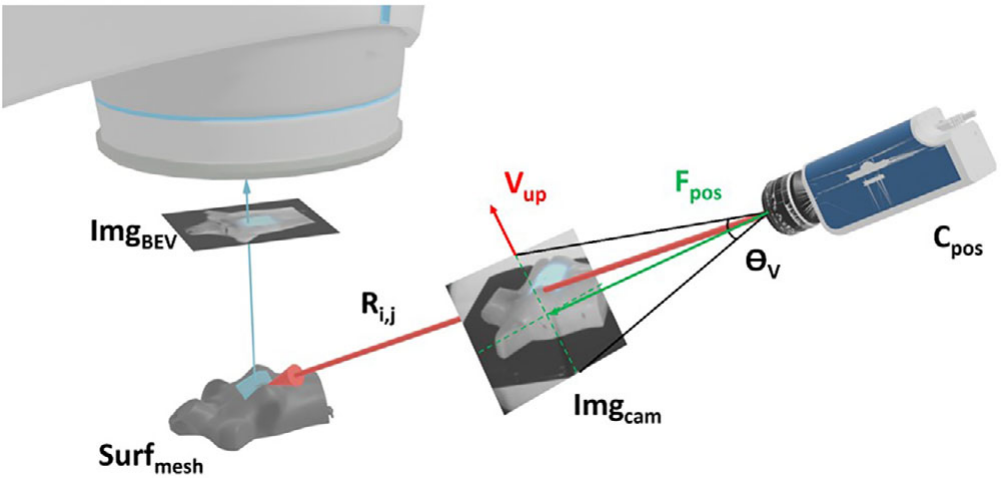
[Raycasting algorithm] Raycasting algorithm visualization, showing the key variables needed to transform the image from the camera perspective to the virtual beam's eye view. Key variables include camera position, field of view, and patient CT surface mesh position.

The following set of equations describes the steps to transform the original image matrix Img_i_ into the beam's eye view projection Img_BEV_ (all bolded variables are matrices/vector). For a visual guide to each variable, see Figure 3. First, to ensure proper texturing resolution at the isocenter approximately 1.4 m from the camera, the Cherenkov images were up‐sampled by a factor of 3 to generate an effective resolution of 5760 × 3600. To generate the rays to be cast from the image onto the patient surface mesh Surf_mesh_, the essential parameters are the camera position (treated as a point source) C_pos_, the camera focal position vector F_pos_, the view up vector that describes the correct image orientation V_up_, and the view angle that describes the angular field of view ϴ_v_. To transform the original two‐dimensional image in the xy plane Img_i_ to the three‐dimensional plane at the correct spacing and orientation from the camera Img_cam_, the following matrix operations were performed

(1) Imgcam=Imgix,y,0Rottot+Cpos

 Where Rot_tot_ is the rotation matrix calculated from the corresponding quaternion representation to transform the flat imaging plane to the camera's imaging plane position and is calculated as

(2) $$Q_{\mathrm{cam}}\left(w_{1},x_{1},y_{1},z_{1}\right)=\left[1+{\hat{F}}_{\mathrm{pos}}\cdot \hat{V_{i}},{\hat{F}}_{\mathrm{pos}}\times \hat{V_{i}}\right]$$

(3) $$Q_{\mathrm{up}}\left(w_{2},x_{2},y_{2},z_{2}\right)=\left[1+{\hat{V}}_{\mathrm{up}}\cdot \hat{W_{i}},{\hat{V}}_{\mathrm{up}}\times \hat{W_{i}}\right]$$

where V_i_ is the normal vector to the initial image (in this case the vector (0,0,1)) and W_i_ is the view up vector for the initial image (in this case the vector (0,−1,0)). To convert these quaternion notations to the rotation matrix Rot_tot_, the following operations were applied

(4) $$\begin{array}{cc} & \mathrm{Ro}t_{\mathrm{cam}}\\ & \;=\left[\begin{array}{ccc} 2(w_{1}^{2}+x_{1}^{2})-1 & 2\left(x_{1}y_{1}-w_{1}z_{1}\right) & 2\left(x_{1}z_{1}+w_{1}y_{1}\right)\\ 2\left(x_{1}y_{1}+w_{1}z_{1}\right) & 2(w_{1}^{2}+y_{1}^{2})-1 & 2\left(y_{1}z_{1}-w_{1}x_{1}\right)\\ 2\left(x_{1}z_{1}-w_{1}y_{1}\right) & 2\left(y_{1}z_{1}+w_{1}x_{1}\right) & 2(w_{1}^{2}+z_{1}^{2})-1 \end{array}\right] \end{array}$$

(5) $$\begin{array}{cc} & \mathrm{Ro}t_{\mathrm{up}}\\ & \;=\left[\begin{array}{ccc} 2(w_{2}^{2}+x_{2}^{2})-1 & 2\left(x_{2}y_{2}-w_{2}z_{2}\right) & 2\left(x_{2}z_{2}+w_{2}y_{2}\right)\\ 2\left(x_{2}y_{2}+w_{2}z_{2}\right) & 2(w_{2}^{2}+y_{2}^{2})-1 & 2\left(y_{2}z_{2}-w_{2}x_{2}\right)\\ 2\left(x_{2}z_{2}-w_{2}y_{2}\right) & 2\left(y_{2}z_{2}+w_{2}x_{2}\right) & 2(w_{2}^{2}+z_{2}^{2})-1 \end{array}\right] \end{array}$$

The Rot_cam_ rotation matrix accounts for the rotation of the initial image plane normal vector to the camera's imaging plane normal vector. The Rot_up_ rotation matrix accounts for the rotation of the initial image view up vector the camera's view up vector. These rotation matrices are then multiplied together to generate the full three‐dimensional rotation of the image.

(6) $$\mathrm{Ro}t_{\mathrm{tot}}=\mathrm{Ro}t_{\mathrm{cam}}.\mathrm{Ro}t_{\mathrm{up}}$$

Each pixel in the up sampled Img_cam_ imaging plane denotes a ray that will be cast onto the patient surface mesh Surf_mesh_. Raycasting requires an initial position and a direction. The initial position is the camera position C_pos_ and the direction of the i, jth ray R_i, j_ is the calculated for the i, j pixel of the image as

(7) Ri,j=Imgii,j,0Rottot

Each ray is then cast using Open3D and intersected with the patient surface mesh Surf_mesh_. The rays that intersect the Surf_mesh_ triangular mesh are checked to make sure only the first hit is counted. This set of intersections then forms a pointcloud Int_rays_ of intersection positions and is used to generate the virtual beam's eye view image Img_BEV_ that will be discussed in the next section.

All of these matrix operations and their associated datasets were performed with a Python script to project the Cherenkov image onto the patient surface. The complete list of ancillary libraries can be found in the code appendix.

### 2.3 Virtual beam's eye view (BEV) imaging

**FIGURE 4:**
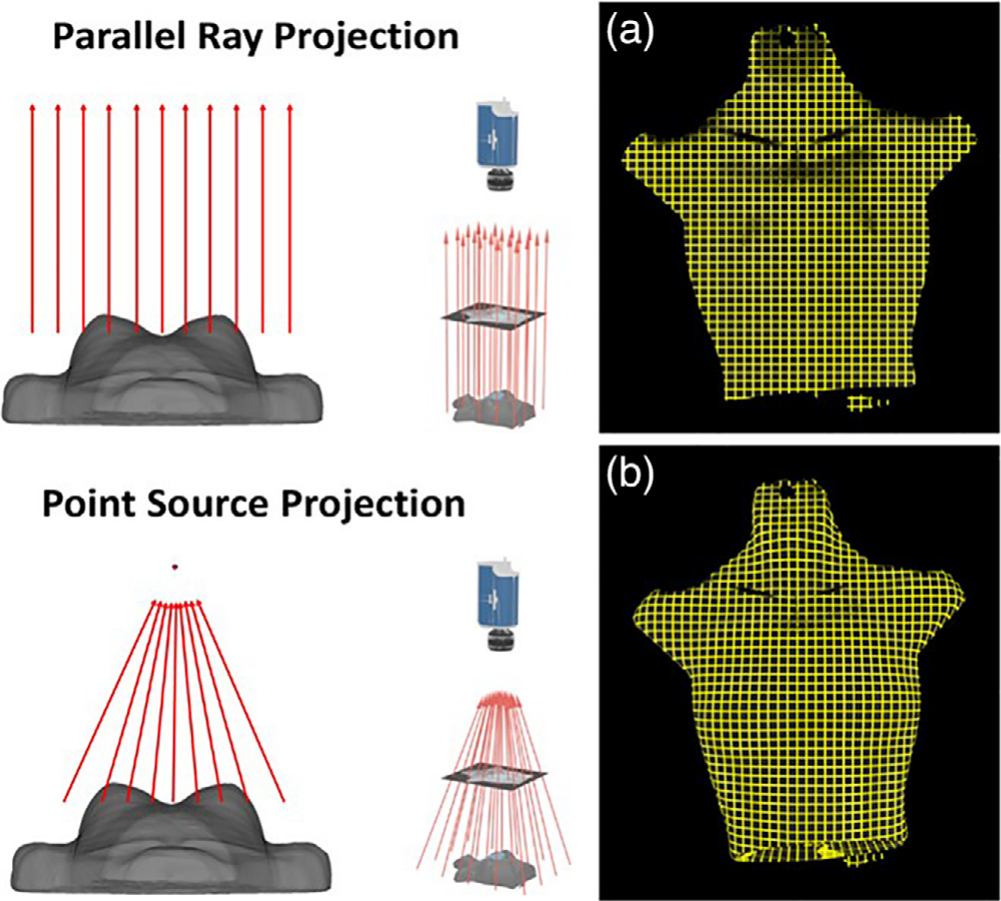
[Virtual beam's eye view imaging] (Parallel ray projection)—Virtual beam's eye view (BEV) Image has uniform spatial resolution, but beam shape differs from MLC shape. Parallel Ray Projection images allows assessment of the spatial distance of radiation fields in 2D images. (Point Source Projection)—Virtual BEV Image matches MLC beam shape, but lacks uniform spatial resolution. (a) shows the beam's eye view image with uniform resolution and no distortion whereas (b) shows spatial barrel distortion.

Virtual beam's eye view images were generated by placing a virtual Cherenkov camera at the beam source position and imaging the Cherenkov‐textured patient surface mesh. The virtual source position was determined geometrically by moving 100 cm from the isocenter in the direction of the gantry. The pointcloud Int_rays_ formed by casting the Cherenkov rays onto the patient surface mesh Surf_mesh_ contains position (x,y,z) and color (R,G,B) data for each ray that intersects the surface. Two types of virtual beam's eye view image Img_BEV_, were formed from this camera perspective (See Figure 4).

1. Point source images Img_BEV_^Point^: Point source projections were created by casting rays from the intersection pointcloud Int_rays_ towards the virtual Cherenkov camera located at the beam source position in the linear accelerator C_pos_^v^ with the same view angle ϴ_v_, to keep the camera image the same size. The rays intersect the virtual imaging plane (see Figure 4) and generate a pointcloud Int_rays_^v^ with position and color for each ray (xyzrgb). To generate a 1920 × 1200 image from this planar pointcloud, it is necessary to first transform Int_rays_^v^ to the XY plane, then sample the transformed pointcloud Img_BEV_^Point^ with the corresponding XY resolution.
(8) $${\mathrm{Img}}_{\mathrm{BEV}}^{\mathrm{Point}}={\mathrm{Int}}_{\mathrm{rays}}^{v}\;{\mathrm{Rot}}_{\mathrm{vtot}}^{-1}-C_{\mathrm{pos}}^{v}$$

Here, Rot_vtot_^−1^ is the inverse of the rotation matrix for the beam's eye view virtual camera position generated the same way as the Cherenkov camera position but using the virtual camera position C_pos_^v^, the virtual focal position F_pos_^v^, the virtual view up vector V_up_^v^, and the view angle ϴ_v_.

To convert the XY plane pointcloud Img_BEV_^Point^ to a 2D image of the same size as the original Cherenkov image, the plane is divided into a 1920 × 1200 matrix. Within each matrix subsection, ray intersections are checked. If no points exist, then color of the pixel is black. If one or multiple rays exist, RGB values are averaged to produce the pixel value.

This method provides the image that one sees in a Beam's Eye View as seen in Eclipse or other treatment planning systems. The advantage of this type of beam's eye view image is that the observed Cherenkov beam outline resembles that of the multi‐leaf collimator (MLC) outline when viewed from this point of view. The drawback of this beam's eye view image is that the divergence of the rays means that each pixel does not denote a fixed distance in x and y, making spatial measurements difficult on this image.

2. Parallel ray images Img_BEV_^Parallel^: Parallel ray projections were created by castings the rays from the patient surface directly along the z axis (from the table to the treatment head) towards the virtual Cherenkov camera located at the beam source position in the linear accelerator. This is equivalent to rotating the intersection pointcloud Int_rays_ to the XY plane and then eliminating the z component of the XYZRGB dataset.
(9) $${\mathrm{Img}}_{\mathrm{BEV}}^{\mathrm{Parallel}}=\mathrm{In}t_{\mathrm{rays}}\;{\mathrm{Rot}}_{\mathrm{vtot}}^{-1}$$
  Here, Rot_vtot_^−1^ is the inverse of the rotation matrix for the beam's eye view virtual camera position as described above. To transform the Img_BEV_^Parallel^ pointcloud in the XY plane to a 2D image, the image plane is again sampled, but this time with a resolution that corresponds to a spatial value. A sampling resolution of 0.25 mm per pixel was selected as it allows us to distinguish 0.5 mm objects while retaining sufficient signal in each pixel bin. RGB values in each bin are again averaged as before and used as the pixel value.

This method ensures that 1 pixel in the image corresponds to a fixed distance in the imaging plane (0.25 mm). The benefit of the parallel ray projection is that beam outline positions can be measured in absolute units. For example, two beam outlines can be compared and the overall shift can be determined in units of distance in the imaging plane. The drawback to the parallel ray projection is that beams may not appear similar as the jaw and MLC outlines because of the parallax generated by surfaces extending out of the treatment table towards the beam source position.

### 2.4 Cherenkov camera combination

Combining multiple Cherenkov camera images onto a patient surface improves the coverage of surface Cherenkov detection especially in cases of partial obstruction of one camera due to patient anatomy or gantry occlusions. Each Cherenkov image was independently projected onto the patient surface mesh in Open3D. Data were saved in pointcloud format to prevent one Cherenkov image from overwriting another when both cameras viewed the same section of the patient surface. Virtual imaging of the fused pointcloud was accomplished by taking median pointcloud values for each pixel of the virtual image. Median values were selected because they preserved image intensity between surfaces covered by multiple camera angles. To quantify the advantage of camera combination, the total number of triangles on the patient surface mesh that were hit with rays from both cameras was measured and compared to one camera.

### 2.5 Virtual beam's eye view quantitative image analysis

This study aimed to demonstrate the quantitative assessment of the spatial beam deviations between initial treatment sessions and subsequent fractions. To do this, shifts to the patient were simulated and various metrics (see Table 1) were applied to the virtual beam's eye view images to calculate the overall beam shift. Parallel ray projection virtual beam's eye view images were quantitatively analyzed with two metrics to calculate the beam shift: Hausdorff distance and Iterative Closest Point (ICP).

**TABLE 1:** Beam's eye view metrics.

Method	Equation	Description
Hausdorff Distance	H(Ref,Target)=max(mind(x,y))	Maximum distance between nearest points
	x∈Ref,y∈Target	
Center of Mass	$\mathrm{COM}=\frac{1}{N}\sum_{i=1}^{N}p_{i}$	Average position of points
Translation	$T_{\mathrm{pixel}}=\mathrm{CO}M_{\mathrm{Ref}}-\mathrm{CO}M_{\mathrm{Target}}$	Pixel and millimeter‐level translation
	$T_{\mathrm{mm}}=\frac{T_{\mathrm{pixel}}}{S}$	

Here, the Hausdorff distance is the maximum of the minimum distances between any two pixels in the beam threshold outline where Ref is the reference beam's eye view image with no shifts applied and Target is the shifted beam's eye view image. For the ICP Center of Mass metric, the center of mass for each Target beam outline is calculated and compared to the Ref beam outline. This shift T_pixel_ is then converted from pixels to mm by dividing by the scale factor S to generate the shift in mm, T_mm_. This scaling factor S is selected to be 4 pixels/mm given that the rough size of one pixel at the field of view of the camera is about 0.08 mm. Thus, sampling at 4 pixels/mm or 0.25 mm/px is three times this frequency and therefore higher than the Nyquist frequency and allows us to generate sufficient signal in each pixel bin (see Section 2.3 on Virtual Beam's Eye View Imaging).

To test the accuracy of each metric, a 6MV, 200 MU 10 × 10 cm^2^ field was delivered to the DoseOptics ABS flat phantom. The table was then moved a known amount to simulate an interfraction patient shift and the 6MV 10 × 10 cm^2^ field was redelivered. To correctly account for this shift in the projection, the patient CT surface was shifted by the same amount as the treatment table. The treatment table accuracy was verified in the positional accuracy test (see Results). By applying the metrics to the non‐shifted and shifted images virtual beam's eye view images, a spatial shift vector was generated in mm. The shift vector was then compared to the known table shift to assess the spatial accuracy of the system. To asses X, Y, and Z accuracy, shifts between −5 cm and +5 cm were performed in 1 cm increments, and XYZ shifts between −5 and 5 mm were delivered in 1 mm increments. These measurements were repeated with the anthropomorphic phantom to assess how these metrics performed on a phantom with more complex geometry.

### 2.6 Assessing positional accuracy

Patient surface mesh textures generated with raycasting were verified against a known calibration pattern on a flat phantom to ensure that camera projection was accurately mapped to the patient surface. To do this, a flat Acrylonitrile Butadiene Styrene (ABS) plastic slab (40 cm × 40 cm × 1 cm) phantom with a known 4 × 11 circle pattern was used. This ABS phantom was selected because its material properties mimic water. Projection from one camera was performed to verify the accuracy of the algorithm. A centroid detection algorithm using the Hough transform was utilized to compare the known centroid locations to the projected centroid locations.

### 2.7 Cherenkov beam outline thresholding

Before measuring Cherenkov beam shifts from the virtual beam's eye view, it was imperative to verify that the beam outlines were robust to different thresholds. To verify that this was the case, a variety of shifted 10 × 10 cm^2^ fields were delivered and the corresponding Hausdorff and ICP metrics were plotted as a function of beam threshold intensity. To further strengthen the repeatability and robustness of the metrics for beam position accuracy as a function of threshold chosen, a window of 3% centered at a threshold of 50% of the maximum beam image intensity was selected. Hausdorff and ICP metrics were then calculated on all seven of these threshold images and their median distance from the set of seven threshold levels (47%–53% in 1% increments) was used to remove any potential outliers.

## 3 RESULTS

### 3.1 Camera combination and beam's eye view

**FIGURE 5:**
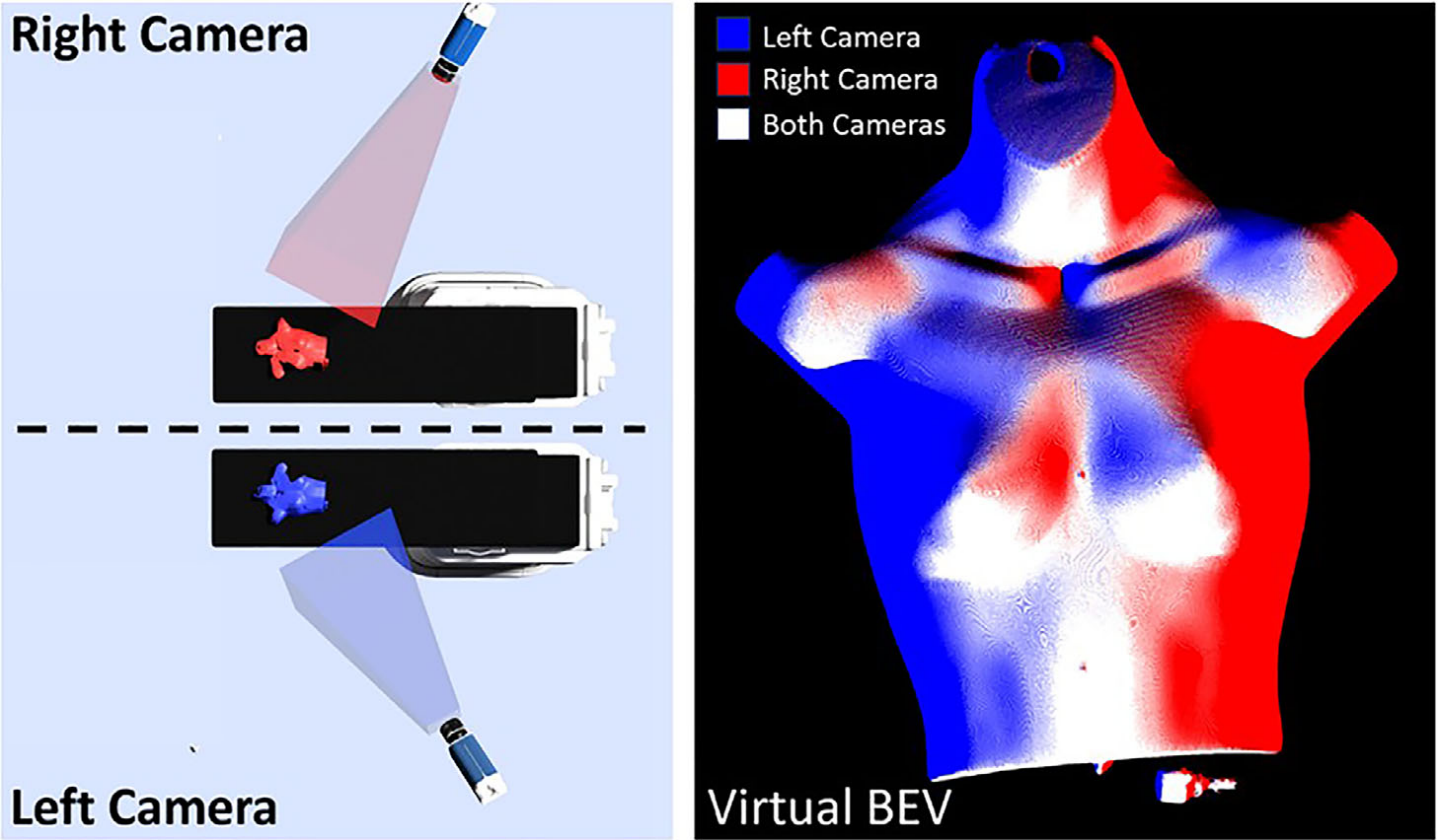
[Patient surface mesh camera coverage] (Left)—Right and Left camera views of the anatomical Annie Phantom surface mesh. (Right) Virtual beam's eye view image with color coding to indicate which camera sees the subsection of each patient surface mesh. The white areas had coverage from both cameras.

Figure 5 shows the surface coverage from each camera (Right camera in Red, Left camera in Blue) and their combined coverage (White). Due to anatomical occlusions of this particular anthropomorphic phantom, both the left and right cameras provided 67% surface coverage. By combining the camera angles, surface coverage of 93% of the phantom CT surface was achieved.

**FIGURE 6:**
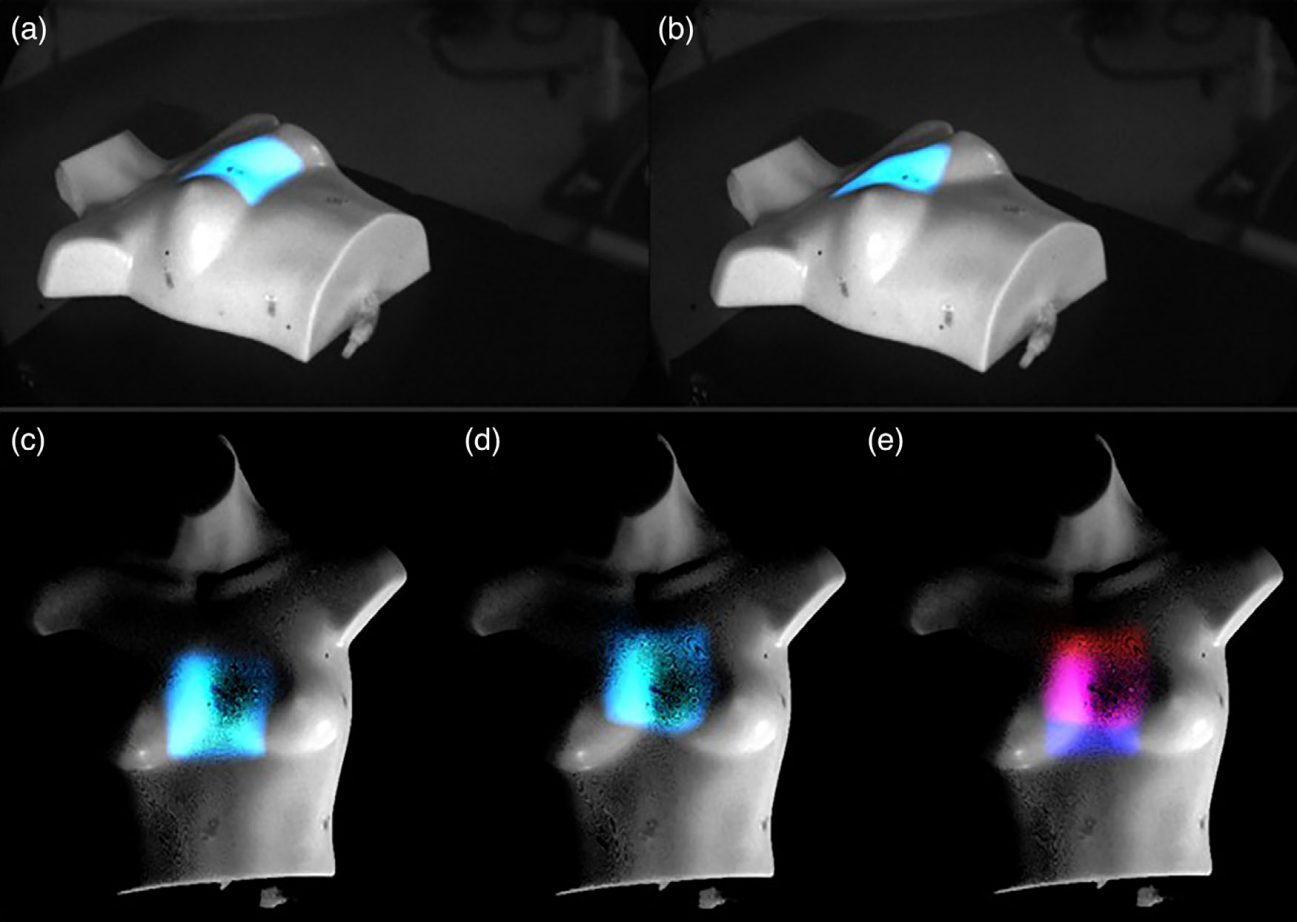
[Beam's eye view imaging in practice] (a) and (b): Two 10 × 10 fields delivered to a phantom. Oblique angle makes it hard to see if the beam shape has changed, or if the beam is shifted. Therapists have no clear way of knowing which it is, and no way of quantifying the difference between the two images. Beam's eye view images (c) and (d) show that the fields are the same shape and (e) shows their overlay, allowing clinical teams to measure their shift in mm.

Camera combination allows for the beam's eye view to be reconstructed while retaining the original beam shape. Figure 6 shows two different fields visualized from an oblique angle, where it is hard to determine if beam shape has changed or if the patient has been shifted. Beam's eye view comparison of the two fields clearly shows that the beam shape is the same, but the beam has shifted significantly. While this may seem trivial for square field shapes, for conformal MLC shapes, the differences are less obvious and the beam's eye view could enable radiation therapists to identify potential treatment incidents much more efficiently.

### 3.2 Positional verification results

**FIGURE 7:**
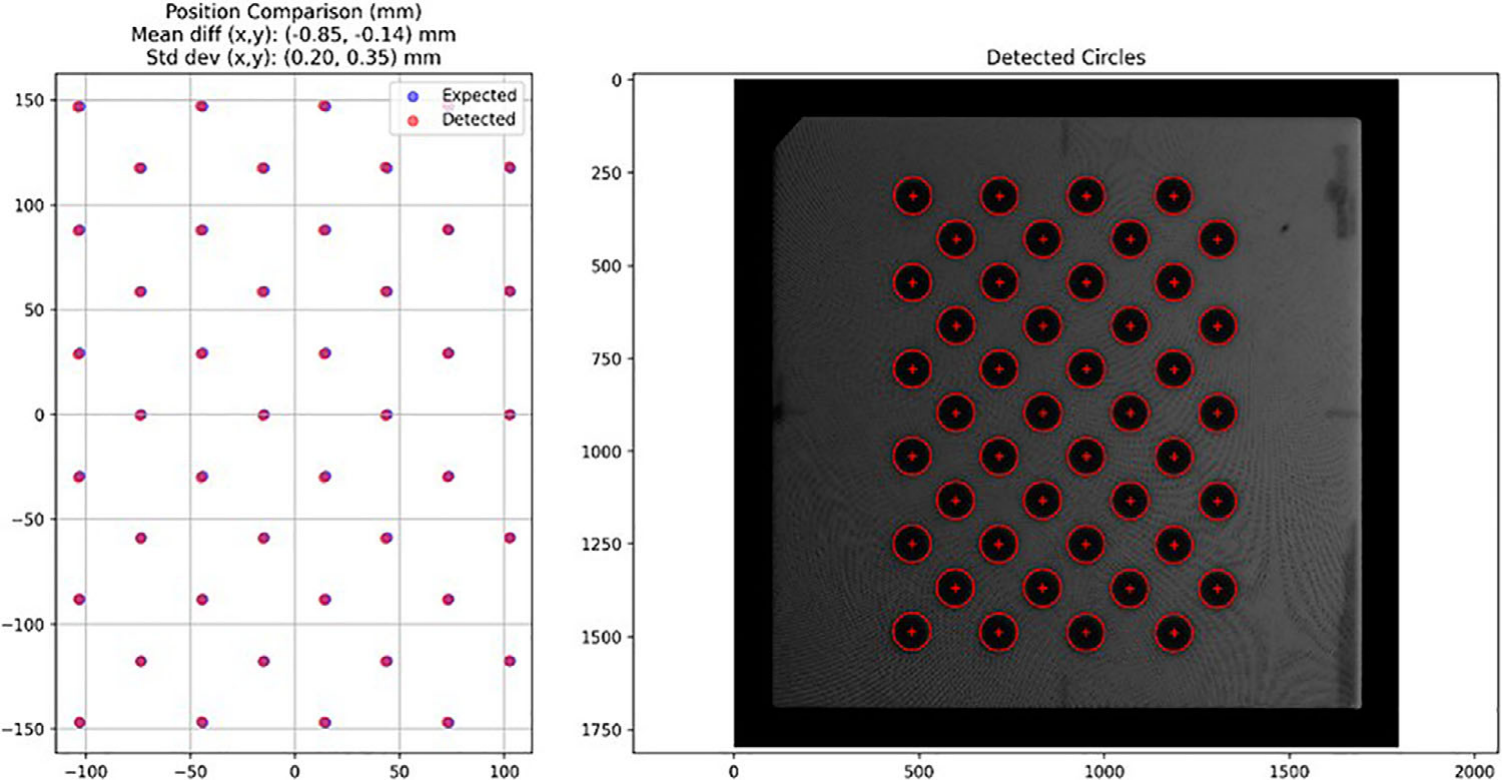
[Projection accuracy validation] Raycasting positional accuracy of beam's eye view images using a DoseOptics flat board phantom. Left: Comparison of detected centroid locations to known locations. Right: detected circles on a calibration phantom. Projected centroid accuracy in x and y is −0.9 mm ± 0.2 mm and −0.1 mm ± 0.4 mm, respectively.

Figure 7 shows the results of the single camera positional verification test. A mean positional setup deviation of 0.85 mm or less was observed with a standard deviation of 0.35 mm or less. Given that the 1920 × 1200 resolution allows for an approximate spatial resolution of around 0.3–0.5 mm depending on the angle of the phantom with respect to the camera, these results demonstrate that the algorithm can properly map the camera images onto the surface meshes within the approximate resolution of the imaging system.

### 3.3 Cherenkov beam outline threshold validation

Both metrics (Hausdorff and ICP) demonstrated high levels of robustness in threshold ranges from 47% to 53% of the maximum intensity with less than 1% deviation. This gives us confidence that these metrics can be used to accurately determine geometric shifts of beam outlines.

### 3.4 Cherenkov beam metric evaluation

**FIGURE 8:**
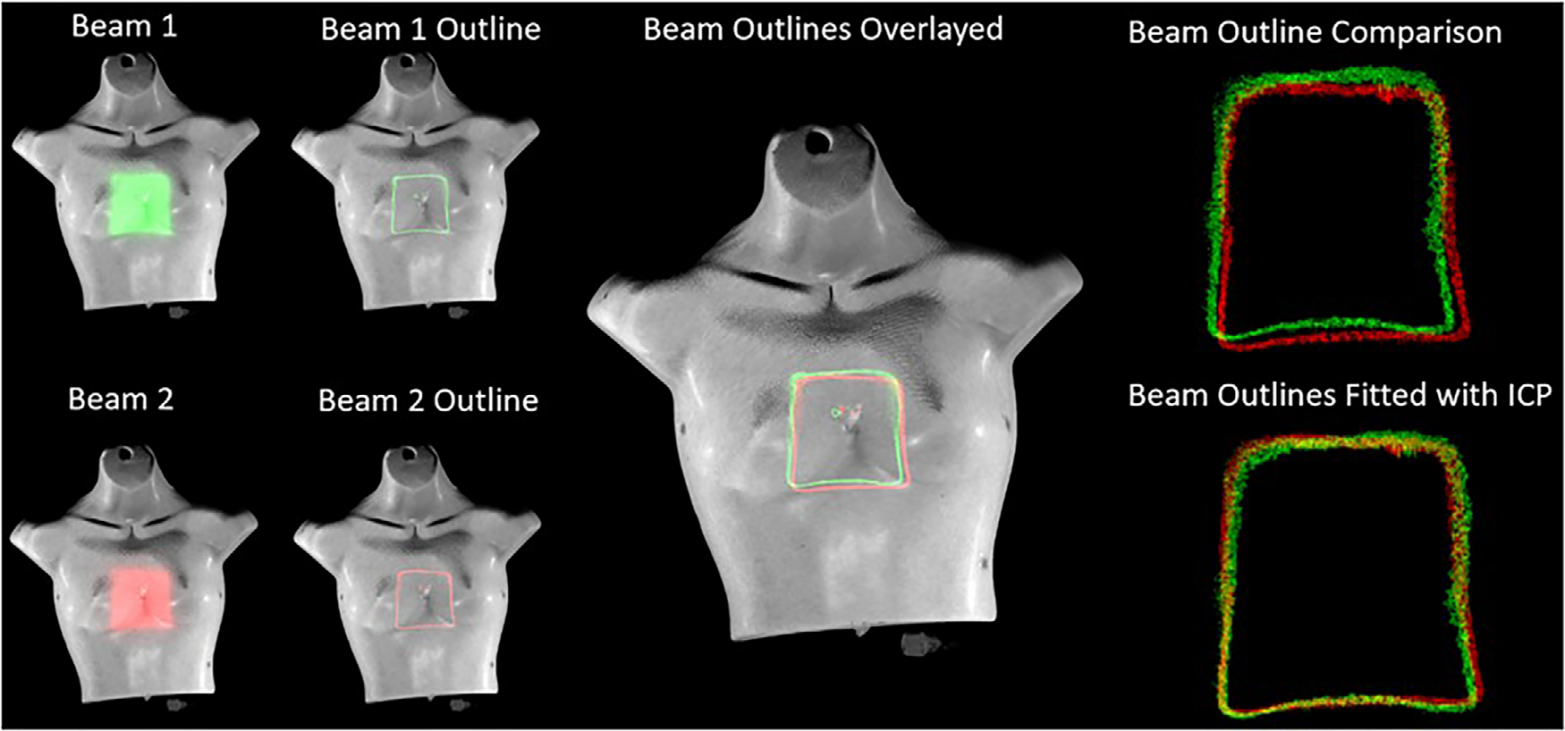
[Cherenkov beam outlines] Beam's eye projections from different fractions. Entire 10 × 10 cm^2^ fields are thresholded to produce beam outlines. The beam outlines are then compared in parallel projection images with uniform spatial resolution of 0.25 mm. Beam metrics calculate shifts and beam outlines are compared.

**FIGURE 9:**
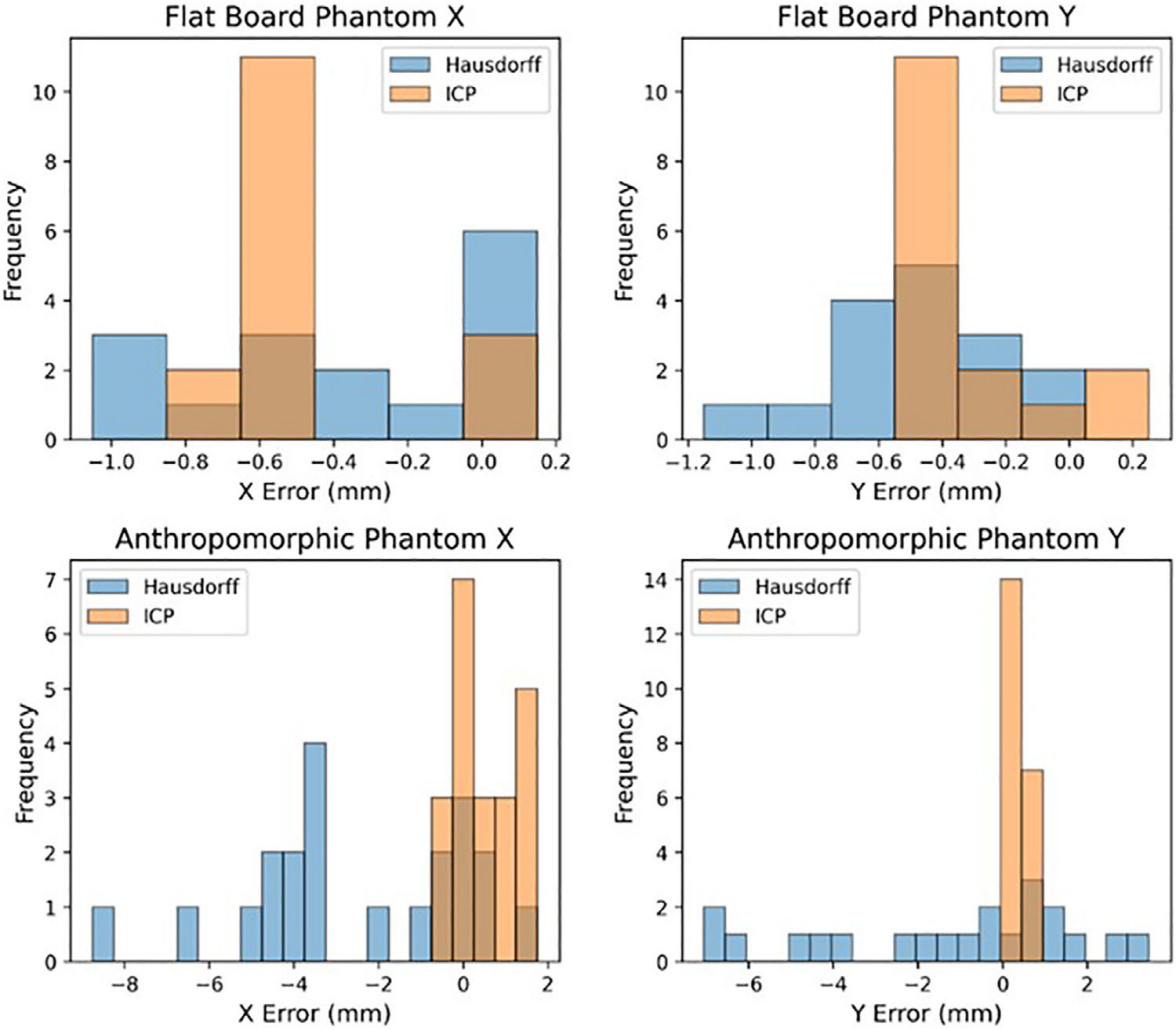
[Spatial beam metrics] ICP (Orange) and Hausdorff (Blue) X and Y spatial error histograms for mm beam shifts on the Flat Board Phantom and Anthropomorphic Annie Phantom. Iterative Closest Point algorithms achieved spatial accuracy under 0.5 mm on both phantoms. Hausdorff metrics achieved spatial accuracy under 0.5 mm on the flat phantom but suffered on the anatomical phantom.

1. Hausdorff distance: Hausdorff distances were calculated for each shift in pixels and converted to mm using the image resolution scaling factor of 4 pixels/mm. Figure 8 shows examples of Cherenkov beam outlines used for spatial metrics. Results (Figure 9) show that Hausdorff distances correspond well with the known geometric shifts on the flat phantoms but suffered on the anatomical Annie phantom due to surface variation. This is because the Hausdorff distance relies on a uniform shift for each corresponding point and surface variations on the Annie phantom mean that this metric is not as stable when compared to the flat phantom. On flat phantoms, the Hausdorff distance metric achieved an average combined x and y accuracy of 0.4 mm ± 0.4 mm and 0.5 mm ± 0.3 mm, respectively. The maximum observed error was 1.1 mm in x and 1.2 mm in y. On the anatomical Annie phantom, the Hausdorff distance metric achieved an average combined x and y accuracy of 2.6 mm ± 2.7 mm and 1.4 mm ± 3.0 mm, respectively. As expected, the accuracy was significantly reduced and errors of up to 8.8 mm were observed compared to ground truth due to the surface deviations present in the Annie phantom and not in the flat phantom.
2. ICP distance: The center of mass ICP metric eliminates some of the shortcoming of the Hausdorff distance by weighing each point, not just one extremum. Because the outlier points from the surface deviations would no longer be so heavily weighted, this metric was expected to perform much better on the anthropomorphic Annie phantom. On the flat phantom, ICP with center of mass achieved an average combined x and y accuracy of 0.5 mm ± 0.2 mm and 0.3 mm ± 0.2 mm, respectively, which was right on par with Hausdorff accuracy. However, ICP achieved lower maximum errors in x and y of 0.7 mm and 0.5 mm, respectively, further demonstrating the increased stability of this metric. On the Annie phantom, ICP achieved an average combined x and y accuracy of 0.5 mm ± 0.7 mm and 0.3 mm ± 0.2 mm, respectively. In addition to a massive improvement in spatial accuracy over Hausdorff, ICP also achieved maximum errors in x and y of 1.6 mm and 0.6 mm, respectively. Not only did ICP achieve substantial performance improvements, but even in the worst case, it did not produce an error of more than 2 mm, which meets the monthly QA guidelines of TG 142.^11^

## 4 DISCUSSION

This work presents the first spatial mapping of Cherenkov into the patient coordinate system, which can be used in real‐time optical verification for EBRT delivery, addressing a critical gap in current treatment verification logistics. While existing verification methods such as CBCT and portal imaging primarily focus on pre‐treatment patient positioning, Cherenkov surface imaging system provides direct visualization and quantitative assessment of actual beam delivery during treatment. This fundamental shift from positional verification to real‐time beam verification represents a significant advancement in radiation therapy quality assurance.

Current clinical practice relies heavily on pre‐treatment imaging and patient setup verification, combined with post‐treatment analysis, creating a temporal gap during in which delivery errors may occur undetected. With weekly chart checks, it is possible that multiple fractions can be delivered with the same spatial errors for many days in a row, prior to being discovered.^12^ Real‐time Cherenkov surface imaging fills this critical safety window by providing continuous monitoring of beam delivery. Qualitative spatial maps provided by Cherenkov images have already proven to be helpful tools for assessing plan delivery^13^ and patient positioning.^14^ This work demonstrates the fusion of spatially distinct Cherenkov images with surface topology, enabling the use of quantitative metrics to assess beam outline deviation between fractions.

The implementation of automated error detection thresholds with high spatial resolution represents a paradigm shift from reactive to proactive quality management. Rather than relying on human vigilance to detect delivery anomalies,^2^ this system has the potential to automatically identify deviations and alert clinical staff in real‐time. While SGRT systems are able to detect potential positioning errors in real‐time, Cherenkov surface imaging will be able to detect beam deviations and quantify their impact. This capability becomes increasingly important as radiation therapy moves toward hypofractionated treatment regimens, where higher doses per fraction increase the clinical consequences of delivery errors.

The multi‐camera integration demonstrated here overcomes the occlusion limitations that have historically constrained optical monitoring systems and offers significant clinical advantages. Achieving 93% surface coverage through camera combination ensures comprehensive monitoring for most patient geometries and treatment angles. This coverage is essential for detecting localized delivery errors that might be missed by single‐viewpoint systems or point‐dose verification methods. However, the major innovation is the ability to view the treatment as a 3D surface from any perspective. Not only does Cherenkov surface imaging introduce the potential to remove gantry occlusions for treatments such as VMAT,^15^ but it allows clinical staff the ability to quantitatively measure Cherenkov image deviations on the patient's surface. This is a key step in the direction towards the ultimate goal of optical on‐patient dosimetry^16^ with Cherenkov imaging as for the first time it gives three‐dimensional coordinates to the Cherenkov intensity map.

The integration potential with surface‐guided radiotherapy (SGRT)^17^ systems present particularly promising opportunities. Because patient surfaces can move during treatment delivery, using rigid CT surfaces does not represent the patient surface as accurately as a live patient surface. While this work demonstrated an approach using static CT‐derived surface meshes, the algorithmic framework readily accommodates real‐time SGRT surface data. This integration would enable dynamic tracking of both patient motion and beam delivery, providing a comprehensive real‐time verification system that addresses both geometric and dosimetric accuracy. Future work will aim to generate Cherenkov surface images on SGRT surfaces^18^ to more accurately represent real‐time patient surface anatomy. In combination with scintillator mesh techniques with the ability to provide dosimetric information in key treatment regions such as match lines, Cherenkov surface imaging would be able to combine positional and dosimetric accuracy on the patient surface to ensure the highest level of real‐time, fully passive, on‐patient dosimetry possible.

## 5 CONCLUSION

This work advanced the capability for spatial assessment of Cherenkov on the patient, with potential for real‐time beam verification, addressing a key step in the simple logistics of treatment delivery accuracy verification. Providing simple intuitive tools for this at the radiotherapy console have challenged the field since the introduction of complex treatment techniques. As treatment delivery becomes increasingly sophisticated with IMRT, VMAT, and emerging adaptive techniques, the need for correspondingly sophisticated verification methods and automation approaches will become more critical. The technique pioneered here provides a scalable verification platform that can evolve with advancing treatment modalities.

The combination of sim‐CT surface placement of Cherenkov images from both lateral cameras, provided sub‐millimeter accuracy, and was implemented in an algorithm that is sufficiently fast for real‐time capability. The approach can be passively implemented, and the result is an intuitive visualization that the therapy beam needs to make fast decisions at the console.

Rather than replacing existing verification methods, this system complements current approaches by providing real‐time capability that existing methods currently do not offer. This complementary role enhances the overall robustness of treatment verification without disrupting established clinical workflows while making a crucial step towards automatic incident detection and on‐patient optical dosimetry.

## AUTHOR CONTRIBUTIONS

Alexander Geiersbach and Petr Bruza developed and tested the Cherenkov surface imaging system. Michael Jermyn assisted with 3D and camera data proesssing. David Gladstone, Lesley Jarvis, Brian Pogue, and Petr Bruza supervised the work and contributed to the manuscript revision.

## CONFLICT OF INTEREST STATEMENT

Authors Brian Pogue, Michael Jermyn, and Petr Bruza are affiliated with DoseOptics LLC, who provided hardware support for this study at no cost.

## Data Availability

Research data will be shared upon reasonable request to the corresponding author.
